# Supplementary material for: What Is the Impact of Intraoperative Microscope-Integrated OCT in Ophthalmic Surgery? Relevant Applications and Outcomes. A Systematic Review
Source: J Clin Med. 2020 Jun 2;9(6):1682. doi: 10.3390/jcm9061682 (PMC7356858; doi:10.3390/jcm9061682)
Supplement: Supplementary file 1 [file jcm-09-01682-s001.zip › Table S1.docx]

**Table 1. Anterior segment studies included**

**Glaucoma surgery**

| Author | Year | Study Design | Sample | MI-OCT | Ocular assessments | Outcomes | Level | Strength | Grade |
| --- | --- | --- | --- | --- | --- | --- | --- | --- | --- |
| Junker B | 2017 | Retrospective case series | 9 | Rescan 700, Carl Zeiss, Meditec | To evaluate intraoperatively success of ab interno trabeculotomy | MI-OCT objectifies trabecular meshwork removal | 5 | III | Low |
| Tanito M | 2017 | Retrospective case series | 9 | Rescan 700, Carl Zeiss, Meditec | Feasibility assessment of MI-OCT during microhook ab interno trabeculotomy | -Images obtained in 83% of cases;  -Classification of the trabeculotomy cleft in 3 patterns: anterior, middle and posterior opening | 4 | III | Low |
| Dada T | 2016 | Retrospective case series | 2 | Rescan 700, Carl Zeiss, Meditec | Bleb revision evaluation with MI-OCT | Detailed view of bleb wall and fibrosis | 4 | III | Very low |
| Siebelmann S | 2016 | Case report | 1 | Rescan 700, Carl Zeiss, Meditec | Canaloplasty evaluation with MI-OCT | Evaluation of Schlemm’s canal and suture tension | 4 | III | Very low |
| Kumar RS | 2015 | Prospective case series | 4 | Rescan 700, Carl Zeiss, Meditec | Demonstrate the use of a spectral-domain MI-OCT in glaucoma surgery | MI-OCT was feasible in all cases | 5 | III | Low |
| Heindl LM | 2015 | Prospective case series | 2 | MI-OCT prototype | MI-OCT in ab interno trabeculotomy | Visualization of chamber angle and trabecular meshwork | 5 | III | Very low |

**Cataract surgery**

| Author | Year | Study Design | Sample | MI-OCT | Ocular assessments | Outcomes | Level | Strength | Grade |
| --- | --- | --- | --- | --- | --- | --- | --- | --- | --- |
| Anisimova NS | 2020 | Retrospective study | 28 | Rescan 700; Carl Zeiss Meditec | MI-OCT during cataract surgery to confirm the presence of incomplete vitreolenticular adhesion | Identification of Berger’s space in 75 % of cases | 4 | III | Very low |
| Titiyal JS | 2020 | Prospective study | 50 | Rescan 700; Carl Zeiss Meditec | Evaluate morphological characteristics and intraoperative dynamics of different type of white cataract with MI-OCT | -Identification of four type of white cataract;  -Prevent rhexis-related complications. | 4 | III | Low |
| Sahay P | 2019 | Case report | 1 | MI-OCT prototype | MI-OCT assisted lens removal in patient affected by ectopia lentis removal | MI-OCT guided lens aspiration | 5 | III | Very low |
| Titiyal JS | 2018 | Prospective study | 129 | Rescan 700; Carl Zeiss Meditec | Morphology of clear corneal incisions and its impact on incision-site Descemet’ membrane detachment in conventional phacoemulsification and femtosecond laser-assisted cataract surgery | Ragged morphology of the incision in 87,1% of cases was related to Descemet’ membrane detachment | 4 | III | Low |
| Titiyal JS | 2017 | Prospective study | 40 | Rescan 700, Carl Zeiss, Meditec | MI OCT evaluation of intraoperative vaulting in patients undergoing implantable collamer lens implantation | -Intraoperative vaulting correlates with postoperative vaulting;  -Improved safety | 4 | III | Low |
| Lytvynchuk LM | 2016 | Prospective study | 74(101 eyes) | Rescan 700, Carl Zeiss, Meditec | MI-OCT assessment of intraocular lenses position at the end of standard phacoemulsification | The mean distance between the IOL central optic and the posterior capsule was 0.71 pixel | 4 | III | Low |
| Das S | 2016 | Prospective study | 38 | Rescan 700, Carl Zeiss, Meditec | Use and application of MI-OCT in micro incision cataract surgery and femtosecond laser assisted cataract surgery | -Assess wound morphology;  -Decide the adequate depth of trenching;  -Position of IOL | 4 | III | Low |
| Pfau M | 2016 | Retrospective study | 9 | Rescan 700, Carl Zeiss, Meditec | Initial clinical experience with the first commercially available MI-OCT system | -Additional information in 22,2% of cases;  -No cases of altered decision-making  -Imaging time 167 seconds | 4 | III | Low |
| Ehlers JP | 2014 | Prospective study | 10 | Rescan 700, Carl Zeiss Meditec | To assess the feasibility and effect on surgical decision making of a MI-OCT DISCOVER study preliminary results | MI-OCT evaluated corneal incisions, scleral closure, phacoemulsification groove depth and IOL position | 4 | III | Low |

**Cornea surgery**

| Author | Year | Study Design | Sample | MI-OCT | Ocular assessments | Outcomes | Level | Strength | Grade |
| --- | --- | --- | --- | --- | --- | --- | --- | --- | --- |
| Muijzer MB | 2020 | Prospective study | 38 | Rescan 700; Carl Zeiss Meditec | MI-OCT for surgical safety, efficiency and outcome in DMEK | -MI-OCT helped surgical decision making in 43% of cases  -Surgery time was shorter using brief over pressurization (44,41±11,61 minutes) | 4 | III | Very low |
| Sharma N | 2020 | Prospective study | 25 | Rescan 700; Carl Zeiss Meditec | MI-OCT for performing DMEK in corneas with poor visualization | -Complete graft attachment in 72% of cases  -Graft orientation was correctly identified in all cases  -16% of cases graft detachment was noted and required rebubbling  -Visual acuity improvement in all cases  - Endothelial cell loss was 36% at 6 months | 4 | III | Very low |
| Patel AS | 2020 | Prospective study | 100 | Rescan 700; Carl Zeiss Meditec | Utility of MI-OCT based on surgeon reporting during intraoperative graft unscrolling and frequency of postoperative complications. | -43 eyes operated by an expert surgeon; 57 eyes operated by six fellows;  -average unscrolling time 4,4 ± 4,1 minutes;  - graft orientation was verified in the external video monitor;  -9 eyes required rebubbling;  -2 eyes experienced graft failure | 4 | III | Low |
| Siebelmann S | 2019 | Retrospective case series | 2 | iOCT; Haag Streit Surgical | MI-OCT-guided puncture and drainage of intrastromal fluid pockets combined with anterior chamber sulfur hexafluoride-fill and pre-descemetic sutures using a commercially available MI-OCT | -Decreased Descemet’s membrane detachment (case1);  - Improves visual acuity to 20/60 (case2) | 5 | III | Very low |
| Agarwal R | 2019 | Case report | 1 | Rescan 700; Carl Zeiss Meditec | Clinical diagnosis and management of acute corneal hydrops | -Improvement of visual acuity to 3/60  - Disappearance of corneal oedema | 5 | III | Very low |
| Bachmann B | 2019 | Retrospective study | 3 | iOCT, Haag-Streit Surgical | Massive corneal hydrops in acute keratoconus treated by mini DMEK | -All patients improved visual acuity;  -1 failed at first attempt;  -2 patients developed partial graft detachment | 4 | III | Very low |
| Schmidt EM | 2019 | Case report | 1 | Rescan 700; Carl Zeiss Meditec | MI-OCT as a real-time imaging tool to help guide corneal biopsy | Visualize the depth and extraction of a corneal biopsy | 5 | III | Very low |
| Titiyal JS | 2019 | Case report | 1 | Rescan 700, Carl Zeiss, Meditec | Identify a sign to confirm correct graft orientation in ultra-thin DSAEK | Correct graft orientation confirmed by 'acute-angled bevel sign' | 5 | III | Very low |
| Singh A | 2019 | Case report | 1 | Rescan 700; Carl Zeiss Meditec | MI-OCT assisted lamellar corneal transplant in a patient with gelatinous drop like corneal dystrophy | MI-OCT guided manual DALK in presence of stromal scarring | 5 | III | Very low |
| Sharma N | 2018 | Prospective case series | 7 | Rescan 700, Carl Zeiss, Meditec | Ulcer debridement with amniotic membrane transplant under the guidance of continuous intraoperative anterior segment MI-OCT | -Surgery successfully completed in all cases  - No recurrences after 2 months | 4 | III | Very low |
| Ehlers JP | 2018 | Prospective study | 244 | Rescan 700 Carl Zeiss Meditec, Cole Eye Institute iOCT prototype, Leica EnFocus system | To report the 3-year assessment of feasibility and usefulness of MI-OCT in DISCOVER study | MI-OCT altered surgical decision in 43.4% of cases.  123 DSAEK, 59 DMEK and 10 DALK procedures performed | 4 | III | Low |
| Siebelmann S | 2018 | Prospective study | 8 | iOCT,Haag-Streit Surgical | Potential benefits of the intraoperative assessment by microscope-integrated MI-OCT of corneal properties during phototherapeutic keratectomy. | -MI-OCT was possible in all patients;  - Tissue intensity decreased significantly after keratectomy;  -CCT decreased significantly after corneal abrasion | 4 | III | Very low |
| Pujari A | 2018 | Case report | 1 | MI-OCT prototype | MI-OCT in corneal trauma to delineate the extent of the corneal perforation and the associated anterior segment changes | Understanding of wound related problems | 5 | III | Very low |
| Selvan H. | 2018 | Case report | 1 | Rescan 700, Carl Zeiss, Meditec | MI-OCT management of triple chamber in DALK | Viscoelastic was identified as the cause and removed | 5 | III | Very low |
| Petrovic A | 2018 | Case report | 1 | En Focus, Leica | MI OCT management of synechiolysis in penetrating keratoplasty | Real time feedback of the anatomy | 5 | III | Very low |
| Steverink JG | 2017 | Prospective case series | 8 | Rescan 700, Carl Zeiss, Meditec | MI-OCT in evaluating graft adhesion and graft interface in patients undergoing Descemet's stripping in DSAEK | -21 out of 24 images interval were obtained;  - 6 persisting fluid interfaces;  - 1 graft detachment | 5 | III | Very low |
| Eguchi H | 2017 | Case report | 1 | Rescan 700, Carl Zeiss, Meditec | MI-OCT assisted penetrating keratoplasty | -MI-OCT notice irido-corneal adhesion | 5 | III | Very low |
| Urkude J | 2017 | Case report | 1 | Rescan 700, Carl Zeiss, Meditec | Management of a case of cap-lenticule adhesion during small incision lenticule extraction using MI-OCT | Visualization of the lenticule | 5 | III | Very Low |
| Titiyal JS | 2016 | Prospective study | 30 | Rescan 700, Carl Zeiss, Meditec | Evaluation of time to donor lenticule apposition in cases of DSAEK with MI-OCT | Donor lenticule apposition lasted 1 to 3 minutes with combined corneal massage and intracameral positive pressure | 4 | III | Low |
| Sharma N | 2016 | Case report | 1 | Rescan 700, Carl Zeiss, Meditec | MI-OCT management of Descemet’s membrane detachment following DALK | Reattachment of Descemet’s Membrane in presence of corneal oedema | 5 | III | Very low |
| Siebelmann S | 2016 | Retrospective case series | 2 | Rescan 700, Carl Zeiss, Meditec | MI-OCT to monitor Boston keratoprosthesis surgery | Utility in assembly and implantation of the prosthesis | 4 | III | Very low |
| Saad A | 2015 | Prospective study | 14 | Rescan 700, Carl Zeiss, Meditec | MI-OCT evaluation of graft orientation during DMEK | - 100% cases correct graft orientation  - mean unfolding time 6,1±3.0 minutes | 4 | III | Low |
| Pahuja N | 2015 | Prospective study | 11 | Rescan 700, Carl Zeiss, Meditec | Compare the penetration of riboflavin with MI-OCT during cross linking | Average depth of penetration in the epi on group was 149.39 ± 15.63 vs 191.04 ± 32.18 microns in the epi-off group | 4 | III | Very low |
| Cost B | 2015 | Prospective case series | 8 | Rescan 700, Carl Zeiss, Meditec, Cole Eye Institute iOCT prototype | Feasibility and utility of MI-OCT in patients undergoing DALK during DISCOVER study | - 100% cases correct graft orientation;  - Improvement of visual acuity in all cases;  - 100% graft survival rate after 4 months | 4 | III | Low |
| Pasricha ND | 2015 | Retrospective case series | 2 | MI-OCT prototype | MIOCT visualization during DSAEK in patients with advanced bullous keratopathy. | Visualization of the donor graft even in presence of marked corneal oedema | 5 | III | Very low |
| Ehlers JP | 2015 | Prospective study | 91 | Rescan 700 Carl Zeiss Meditec, Cole Eye Institute iOCT prototype | To assess the feasibility and effect on surgical decision making of a MI-OCT DISCOVER study one-year result | MI-OCT was feasible in almost all cases  MI-OCT altered surgical decision in 38% of cases | 4 | III | Low |

**Paediatric examination**

| Author | Year | Study Design | Sample | MI-OCT | Ocular assessments | Outcomes | Level | Strength | Grade |
| --- | --- | --- | --- | --- | --- | --- | --- | --- | --- |
| Coppola M | 2018 | Case report | 1 | Rescan 700; Carl Zeiss Meditec | MI-OCT in paediatric examination | MI-OCT excluded the need of reintervention | 5 | III | Very low |
| Siebelmann S | 2015 | Retrospective case series | 2 | Rescan 700, Carl Zeiss, Meditec | MI-OCT examination of children with anterior segment anomalies | Detailed examination of anterior segment anomalies without surgical interruptions | 4 | III | Very Low |

**Strabismus surgery**

| Author | Year | Study Design | Sample | MI-OCT | Ocular assessments | Outcomes | Level | Strength | Grade |
| --- | --- | --- | --- | --- | --- | --- | --- | --- | --- |
| Pihlblad MS | 2019 | Prospective study | 19 | Rescan 700; Carl Zeiss Meditec | Use of different OCT machines to image rectus muscle insertions in paediatric strabismus patients. | Rectus muscle insertion was imaged in 58-89% of cases | 4 | III | Very Low |

MI- OCT: microscope integrated optical coherence tomography; DMEK: Descemet Membrane Endothelial Keratoplasty, DSAEK: Descemet's Stripping Automated Endothelial Keratoplasty, DALK: deep anterior lamellar keratoplasty; IOL: intra ocular lens. The level and the strength of evidence was defined according to the Oxford Centre for Evidence- Based Medicine (OCEM) 2011 guidelines and the Scottish Intercollegiate Guideline Network (SIGN) assessment system for individual studies as implemented for Preferred Practice Patterns by the American Academy of Ophthalmology respectively [14,15]. The quality of evidence based on the Grading of Recommendations Assessment, Development and Evaluation (GRADE) system was also assessed [16].
