# Supplementary material for: What Is the Impact of Intraoperative Microscope-Integrated OCT in Ophthalmic Surgery? Relevant Applications and Outcomes. A Systematic Review
Source: J Clin Med. 2020 Jun 2;9(6):1682. doi: 10.3390/jcm9061682 (PMC7356858; doi:10.3390/jcm9061682)
Supplement: Supplementary file 1 [file jcm-09-01682-s001.zip › Table S2.docx]

**Table 2. Posterior segment studies included**

**Macular surgery**

| Author | Year | Study Design | Sample | MI-OCT | Ocular assessments | Outcomes | Level | Strength | Grade |
| --- | --- | --- | --- | --- | --- | --- | --- | --- | --- |
| Leisser C | 2020 | Prospective study | 25 | Rescan 700 Carl Zeiss Meditec | Measuring the amounts of transient retinal thickening due to tractional forces during membrane peeling and analyzing possible effects on postoperative retinal function. | -Transient retinal thickening owing to tractional forces during peeling could be observed in 64% of cases  -24% of cases developed new deep microscotomata 3 months after surgery, but among them only two patients had transient retinal thickening during peeling | 4 | III | Low |
| Lorusso M | 2020 | Retrospective study | 29 | Rescan 700 Carl Zeiss Meditec | To assess closure rate and visual outcome of a court of patients with repaired MH confirmed by MI-OCT | -MI-OCT is useful tool for prescribing short-term face-down position after surgery, with high closure rate and no additional complication.  -The execution of an OCT in the immediate postoperative days could be potentially unnecessary. | 4 | III | Very low |
| Inoue M | 2019 | Retrospective study | 22 | Rescan 700, Carl Zeiss Meditec | MI-OCT detects the presence of residual fragments at the edge of the MH | Residual fragment detected with MI-OCT at the edge of the macular hole are predictors of limited postoperative visual improvements | 4 | III | Very low |
| Leisser C | 2019 | Prospective study | 171 | Rescan 700, Carl Zeiss Meditec | To evaluate the rate of neurosensory elevation with resulting subfoveal and extrafoveal hyporeflective zones during membrane peeling using MI-OCT | -Iatrogenic subfoveal and extrafoveal hyporeflective zones were visible only in 7% of cases.  -Clinical outcomes similar between patients with or without these findings | 4 | III | Low |
| Leisser C | 2019 | Prospective study | 30 | Rescan700, Carl Zeiss Meditec, | To evaluate if MI-OCT enables membrane peeling without staining | -MI-OCT allow ERM peeling without staining in a majority of cases  -Chomovitrectomy is superior to MI-OCT for ILM peeling | 4 | III | Low |
| Ehlers JP | 2018 | Prospective study | 593 | Rescan 700 Carl Zeiss Meditec, Cole Eye Institute iOCT prototype, Leica EnFocus system | To report the 3-year assessment of feasibility and usefulness of MI-OCT in DISCOVER study | MI-OCT altered surgical decision in 29.2% of cases.  MI-OCT interfered with the surgical procedure in 6% of cases | 4 | III | Low |
| Borrelli E | 2018 | Prospective case series | 3 | Rescan700, Carl Zeiss Meditec, | To evaluate MI-OCT during MH repairment with ILM inverted flap | MI-OCT confirmed ILM inverted flap position, even after air-fluid exchange | 5 | III | Very low |
| Leisser C | 2018 | Prospective study | 69 | Rescan700, Carl Zeiss Meditec, | To assess risk factors for postoperative intraretinal cystoid changes in a study randomized for balanced salt solution and air-tamponade at the end of surgery | The only risk factor identified for postoperative intraretinal cystoid changes after peeling of idiopathic epiretinal membranes is the presence of preoperative intraretinal cystoid changes | 4 | III | Low |
| Leisser C | 2018 | Prospective study | 41 | Rescan700, Carl Zeiss Meditec, | To compare MI-OCT imaging results to a stand-alone spectral-domain OCT | Preoperative OCT and MI-OCT evaluations showed high intraobserver and interobserver reproducibility for the presence of ERM, lamellar macular hole, and vitreomacular traction. For intraretinal cystoid changes, intraobserver and interobserver reproducibility for both OCTs was rather poor, mainly due to microcystic changes. | 4 | III | Low |
| Kumar V | 2018 | Retrospective study | 25 | Rescan 700, Carl Zeiss Meditec | To describe a novel intraoperative finding during pars plana vitrectomy for macular hole using operating microscope-integrated spectral domain optical coherence tomography that predicts the closure of macular hole. | Hole-door sign is a novel intraoperative finding that predicts postoperative Type-1 closure of macular hole | 4 | III | Very low |
| Kumar A | 2018 | Prospective study | 9 | Rescan 700, Carl Zeiss Meditec | To evaluate MI-OCT-guided traction removal and center-sparing internal limiting membrane peeling | MI-OCT-guided center-sparing ILM peeling helps in complete removal of  traction, resolution of retinoschisis and good functional recovery with low  intraoperative and postoperative complications | 4 | III | Low |
| Sawaguchi S | 2017 | Case report | 1 | Rescan 700, Carl Zeiss Meditec | To report the identification of MH formation using MI-OCT during vitrectomy for vitreomacular traction syndrome | Surgical approach can be modified according to MI-OCT imaging | 5 | III | Very low |
| Uchida A | 2017 | Prospective study | 34 | Rescan 700 Carl Zeiss Meditec, Cole Eye Institute iOCT prototype, En Focus, Leica | To evaluate acute retinal alterations identified by MI-OCT immediately following surgical intervention for vitreoretinal interface disorders | Acute retinal alterations after ILM peeling were visualized at a frequency of less than 10%.  Additional research is needed to understand the clinical impact | 4 | III | Low |
| Kumar A | 2017 | Case report | 1 | Rescan 700, Carl Zeiss Meditec | Macular hole-associated retinal detachment in high myopia treatment MI-OCT assisted | MI-OCT helps to identify vitreoschisis and confirm the position of ILM flaps over the macular hole intraoperatively | 4 | III | Low |
| Leisser C | 2016 | Prospective study | 20 | Rescan 700, Carl Zeiss Meditec | To evaluate the quality of MI-OCT of the posterior hyaloid, ERM, ILM and hyporeflective subfoveal zone | MI-OCT is a valuable tool for intraoperative visualization of the ERM and offers immediate visualization of retinal anatomy during peeling. | 4 | III | Low |
| Moisseiev E | 2016 | Case report | 1 | Rescan 700, Carl Zeiss Meditec | To report a case of MH development a week after vitrectomy for vitreomacular traction | MI-OCT findings provide insight into the role of the ILM in macular hole formation. | 5 | III | Very Low |
| Pfau M | 2016 | Retrospective study | 32 | Rescan 700, Carl Zeiss Meditec | Initial clinical experience with the first commercially available MI-OCT system | -Additional information in 74.1% of cases;  -41.9% cases of altered surgical decision  -Imaging time 167 seconds | 4 | III | Low |
| Kunikata H | 2015 | Retrospective case series | 6 | Rescan 700, Carl Zeiss Meditec | To report MI-OCT-assisted 27 Gauge pars plana vitrectomy | MI-OCT imaging provided excellent intraoperative visualization of retinal tissues without causing significant obstructions to the surgeon. | 5 | III | Very low |
| Falkner-Radler CI | 2015 | Prospective study | 70 | MI-OCT prototype | Feasibility and utility of MI-OCT in macular surgery | MI-OCT allowed ERM peeling in 40% of cases without using retinal dyes | 4 | III | Low |
| Ehlers JP | 2015 | Prospective study | 136 | Rescan 700 Carl Zeiss Meditec, Cole Eye Institute iOCT prototype | To assess the feasibility and effect on surgical decision making of a MI-OCT DISCOVER study one-year result | MI-OCT provide valuable feedback in 71% of cases  MI-OCT altered surgical decision in 19% of cases | 4 | III | Low |
| Ehlers JP | 2014 | Prospective study | 10 | Rescan 700, Carl Zeiss Meditec | To assess the feasibility and effect on surgical decision making of a MI-OCT DISCOVER study preliminary results | MI-OCT evaluated hyaloid release with triamcinolone and completeness of peel in MH, epiretinal membrane, and vitreomacular traction | 4 | III | Very Low |
| Binder S | 2011 | Prospective study | 25 | MI-OCT prototype | To evaluate the feasibility of MI-OCT in a pilot study | MI-OCT is feasible  MI-OCT may improve surgical decision making | 4 | III | Low |

**Retinal detachment surgery**

| Author | Year | Study Design | Sample | MI-OCT | Ocular assessments | Outcomes | Level | Strength | Grade |
| --- | --- | --- | --- | --- | --- | --- | --- | --- | --- |
| Abraham JR | 2020 | Post hoc analysis (retrospective) | 103 | Rescan 700 Carl Zeiss Meditec, Cole Eye Institute iOCT prototype, En Focus, Leica | To evaluating MI-OCT utility and outcomes during RD repair. | -MI-OCT provided valuable feedback in 36% of cases  -MI-OCT altered surgical decision making in 12% of cases  -Reoperation rate 6% in uncomplicated cases and 25% in complicated cases | 4 | III | Low |
| Singh A | 2020 | Retrospective case series | 2 | Rescan 700 Carl Zeiss Meditec | To evaluate the feasibility and utility of MI-OCT in patients undergoing full thickness neurosensory retinal autograft for refractory macular hole associated retinal detachment | MIOCT provides intra-operative visualization of macular holes and provides real-time feedback regarding dimensions of the retinal autograft, thus aiding in, accurate sizing of the graft. | 5 | III | Very low |
| Obeid A | 2019 | Retrospective study | 31 | MI-OCT prototype | Evaluation of residual subfoveal fluid immediately after rhegmatogenous RD repair using MI-OCT | No difference was observed in the amount of residual SFF as measured on MI-OCT during RD repair with pars plana vitrectomy using either direct drainage, drainage retinotomy or perfluoron | 4 | III | Very Low |
| Sundar D | 2018 | Case report | 1 | Rescan 700, Carl Zeiss Meditec | To report a case of MI-OCT guided retinotomy in RD repair | MI-OCT helps surgeon to release retinal traction | 5 | III | Very low |
| Lytvynchuk LM | 2017 | Case report | 1 | Rescan 700, Carl Zeiss Meditec | To report a non-rhegmatogenous RD associated with Morning glory syndrome treatment MI-OCT assisted | Based on MI-OCT findings, RD is caused primarily by the vitreous traction with further possible formation of retinal breaks | 5 | III | Very low |
| Leisser C | 2016 | Prospective study | 10 | Rescan 700, Carl Zeiss Meditec | To evaluate integrity of the ellipsoid zone after perfluoro-n-octane use for reattachment of the central retina in macula-off retinal detachment | Perfluoro-n-octane use seems to be a safe procedure regarding integrity of the ellipsoid zone in cases scheduled for primary vitrectomy for retinal detachment repair. | 4 | III | Very low |
| Toygar O | 2016 | Prospective case series | 9 | iOCT; Haag Streit Surgical | To investigate microanatomical relationships during surgical repair of macula involving rhegmatogenous RD | MI-OCT endorses the strict face-down positioning for at least 24-hours after surgery, even if submacular fluid was not clinically evident | 5 | III | Low |
| Smith AG | 2015 | Case report | 1 | Rescan 700, Carl Zeiss Meditec | To evaluate MI-OCT help in subretinal perfluoron removal | MI-OCT facilitates subretinal perfluoron removal | 5 | III | Very low |

**Diabetic retinopathy surgery**

| Author | Year | Study Design | Sample | MI-OCT | Ocular assessments | Outcomes | Level | Strength | Grade |
| --- | --- | --- | --- | --- | --- | --- | --- | --- | --- |
| Agarwal A | 2020 | Retrospective study | 46 | Rescan 700 Carl Zeiss Meditec | MI-OCT assisted proportional reflux hydrodissection during pars plana vitrectomy in eyes with complex proliferative diabetic retinopathy | Combination of mi-OCT and PRH is useful in complete fibrovascular tissue dissection during PPV for complex PDR cases. | 4 | III | Very low |
| Khan M | 2018 | Prospective study | 81 | Rescan 700 Carl Zeiss Meditec, Cole Eye Institute iOCT prototype, Leica EnFocus system | Feasibility and role of MI-OCT in surgical decision-making during vitreoretinal  surgical interventions for proliferative diabetic retinopathy | -MI-OCT offers a real time and valuable feedback to the surgeon in 50,6% of cases  -MI-OCT alters the surgical plan in 26% of cases | 4 | III | Low |
| Runkle A | 2017 | Prospective study | 50 | En Focus, Leica | To evaluate the feasibility and utility of MI-OCT EnFocus in DISCOVER study | -Images obtained in 92% of cases;  -In 16% of cases MI-OCT altered surgical decision | 4 | III | Low |

**Other retinal procedures**

| Author | Year | Study Design | Sample | MI-OCT | Ocular assessments | Outcomes | Level | Strength | Grade |
| --- | --- | --- | --- | --- | --- | --- | --- | --- | --- |
| Gregori NZ | 2019 | Retrospective case series | 3 | Rescan 700, Carl Zeiss Meditec | To report subretinal gene delivery assisted by MI-OCT | MI-OCT provides real-time feedback to guide viral vector injection and allows better definition of the topographical retinal area that received the treatment as well as detection of complications | 5 | III | Very low |
| Itoh Y | 2019 | Retrospective study | 15 | Rescan700, Carl Zeiss Meditec, | To determine the changes in the foveal architecture before and after ILM peeling with and without fovea-sparing ILM peeling during vitrectomy for high myopic retinoschisis by MI-OCT | Endorsement of after fovea-sparing ILM peeling due to lack of alterations of the foveal architecture and significant improvements in the BCVA | 4 | III | Very low |
| Lam BL | 2019 | Phase 2 clinical trial | 6 | Rescan700, Carl Zeiss Meditec, | MI-OCT assisted gene therapy subretinal delivery assessment in patients affected by Choroideremia | Choroideremia gene therapy delivered with MI-OCT has a good safety profile | 5 | III | Low |
| Kumar JB | 2018 | Prospective study | 74 | Rescan 700 Carl Zeiss Meditec, Cole Eye Institute iOCT prototype | Assessment of feasibility and usefulness of MI-OCT in DISCOVER study for uveitic patients | MI-OCT provided valuable feedback for:  -fluocinolone acetonide implant placement in 11 of 13 eyes (84.6%),  -chorioretinal biopsies in 13 of 16 eyes (81.2%)  -retinal detachment (RD) repairs in 20 of 27 eyes (74.1%) | 4 | III | Low |
| Bruyère E | 2018 | Retrospective study | 22 | Rescan700, Carl Zeiss Meditec, | Feasibility and information provided by MI-OCT during vitreomacular surgery in highly myopic eyes. | MI-OCT could help assess undetected macular openings and otherwise posterior vitreous status and epiretinal structure peeling | 4 | III | Very low |
| Browne AW | 2017 | Prospective case series | 6 | Rescan 700 Carl Zeiss Meditec | To assess the utility of MI-OCT during pars plana vitrectomy with chorioretinal biopsy | -MI-OCT facilitates identification of biopsy sites  -After biopsy, MI-OCT verify biopsy completeness and retinal edges status | 5 | III | Low |
| Yaginuma S | 2017 | Case report | 1 | Rescan 700 Carl Zeiss Meditec | To evaluate the efficacy of MI-OCT during vitrectomy for acute endophthalmitis. | Intraoperative OCT can be useful to examine the structural alterations of the retina in eyes with vitreous opacities that prevent preoperative OCT examinations | 5 | III | Very low |
| Rachitskaya AV | 2016 | Prospective case series | 3 | Rescan 700, Carl Zeiss Meditec | To evaluate the feasibility of MI-OCT in Argus II implantation | MI-OCT provides information about electrode array-retina interface | 5 | III | Very low |

MI-OCT: microscope integrated optical coherence tomography; MH: macular hole, RD: retinal detachment. The level and the strength of evidence was defined according to the Oxford Centre for Evidence- Based Medicine (OCEM) 2011 guidelines and the Scottish Intercollegiate Guideline Network (SIGN) assessment system for individual studies as implemented for Preferred Practice Patterns by the American Academy of Ophthalmology respectively [14,15]. The quality of evidence based on the Grading of Recommendations Assessment, Development and Evaluation (GRADE) system was also assessed [16].
